# Relationship between physical activity and cognitive functioning among older Indian adults

**DOI:** 10.1038/s41598-022-06725-3

**Published:** 2022-02-17

**Authors:** Manish Kumar, Shobhit Srivastava, T. Muhammad

**Affiliations:** International Institute for Population Sciences, Mumbai, Maharashtra India 400088

**Keywords:** Geriatrics, Health policy

## Abstract

In a culturally different and low-resource setting, where lifestyle habits, including dietary pattern and physical activities differ from those in high-income countries, the association between physical activity and cognition is expected to differ. We aimed to investigate the association between physical activity and cognitive functioning after controlling for potential confounders among older adults in India. Furthermore, gender differences in this relationship were analyzed. Using a national-level data from the Longitudinal Ageing Study in India (2017–2018), this paper employed propensity score matching (PSM) approach to examine the association between physical activities and cognitive functioning among Indian older adults. Cognitive impairment was measured through five broad domains (memory, orientation, arithmetic function, executive function, and object naming). We limit our sample to older adults aged 60 + years, and our final dataset contains 31,464 participants (men = 16,366, and women = 15,098). The results indicated that older adults who engaged in frequent physical activity have greater cognitive functioning than older adults without physical activity after adjusting for various individual, health, lifestyle, and household factors. This association holds true for both older men and older women. The results from the PSM revealed that the cognitive function score was increased by 0.98 and 1.32 points for the frequently physically active older men and women population, respectively. The results demonstrate the possible beneficial effects of frequent physical activity on cognitive functioning among older adults. Thus, regular physical activity can be considered as an effective lifestyle factor to promote healthy cognitive aging.

## Introduction

Cognitive functioning usually refers to different mental abilities, including thinking, learning, language, reasoning, attention and concentration, and visuospatial functioning^1^. Several theoretical and empirical studies have reported that cognitive functioning declines with age and the older population is prone to cognition-related issues (i.e., cognitive impairment)^2,3^. With the rapid increase in the aging population, the increase in age-related cognitive decline and dementia, including Alzheimer's disease, has become a crucial health burden^4^. Worldwide, nearly 47 million people living with dementia in 2015^5^, and this figure is expected to increase to 132 million by 2050^6^, and most of these demented people are expected to live in the Asian countries^7^. Notably, India, where dementia is an emerging health issue, has nearly 5.3 million people living with dementia which is expected to triple by 2050^8^. Due to higher proportion of older population who suffers from poor mental health and cognitive decline, India will face tremendous public health and socioeconomic challenges^9^. Moreover, cognitive disabilities do not only affect the individual but also poses a major physical, emotional, and economic burden on the family, health care providers, and society^10,11^.

Pharmaceutical therapies related to dementia treatment are still under-developed. In the absence of a cure, there is an urgent need to identify effective modifiable lifestyle-based strategies and protective factors for reducing the potential risk of dementia. For example, Fratiglioni et al. identified three modifiable lifestyle factors that significantly slow the cognitive decline and prevent dementia: cognitive leisure activity, socially integrated network, and regular physical activity^12^. Multiple systematic reviews have reported that out of these lifestyle factors; regular physical activity has the most protective effect against age-related cognitive decline^13–16^. A meta-analysis of 15 cohort studies based on individuals without dementia with a follow-up period of 1–12 years reported an inverse association between physical activity and cognitive decline^17^. Moreover, several experimental and cross-sectional studies reported that physical activity is associated with better cognitive functioning^18–20^. Physical activity exerts importance in other areas of physical health; in particular, regular participation in physical activities could reduce the risk of various chronic diseases, including stroke, coronary heart disease^21,22^, depression^23^, and diabetes^24^, which are well-recognized high-risk factors for cognitive impairment.

Despite well-established protective effects of physical activity against cognitive dysfunction, the existing literature has shown inconsistent results^25–27^. The biological mechanism for the relationship between physical activities and cognition is still unclear. However, there were some theoretical concepts on how physical activity affects cognition, including modification of structural changes in the brain due to exercises^28,29^, and hippocampal neurogenesis and synaptic plasticity initiated due to physical activity, which stimulates the improvement in the cognitive health^30^.

Notably, most of the studies examining the association between physical activity and cognitive functioning have been conducted in western countries^31,32^; there is a scarcity of scientific knowledge regarding the health benefits of physical activities on cognitive function in Indian older adults. In a culturally different and low-resource setting, where lifestyle habits, including dietary pattern and physical activities differ from those in high-income countries, the association between physical activity and cognition is expected to differ. One recent systematic review conducted in India identified 19 peer-reviewed articles investigating the association of physical activity with at least one mental health outcome, including depression, anxiety, psychosis, stress, self-esteem, and cognitive functioning^33^. A recent study based in the state of Odisha in India aimed to examine the determinants of the various mental health disorders, including anxiety, depression, and cognitive disorders, found that physical activity was inversely associated with these disorders^34^. In India, multiple sub-national-level studies generally lack coherent definitions of cognitive functioning and physical activities^34^. By investigating the role of physical activity in cognition, the present study will help policymakers generate a comprehensive policy framework focused on reducing the risk of development of cognitive impairment, in light of physical activities, at the national level. However, there is a lack of national-level studies dedicated to determine the association between physical activity and cognition.

**Figure 1:**
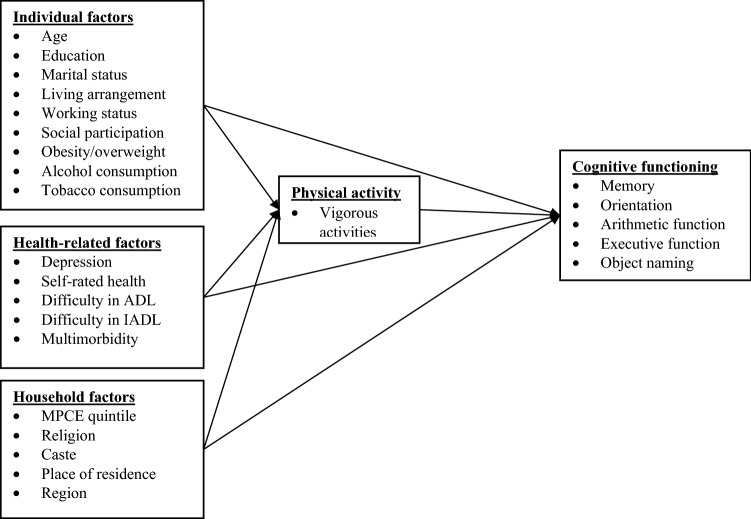
Conceptual framework for cognitive functioning.

By filling this knowledge gap, we aimed to investigate the association between physical activity and cognitive functioning after controlling for potential confounders among older adults aged 60 years and over using the propensity score matching (PSM) based on the data from a large national-level Longitudinal Ageing Study in India (LASI). In addition, considering the potential effect that gender differences pose on both cognition and physical activity, gender differences in this relationship were analyzed. Based on the abovementioned background, we developed a conceptual framework has been summarised in Fig. 1.

## Methods

### Data

The present study is based on the first wave of the Longitudinal Aging Study in India (LASI) conducted during 2017–2018^35^. LASI is a national representative longitudinal survey of middle-and older-aged Indians (i.e., aged 45 years or older) and their spouses who reside in the same households, irrespective of age. The LASI provides rich information on demographics, morbidity, health behavior factors, and physical health of the aging population in India^35^. The LASI survey adopted a multistage stratified area probability cluster sampling design. It is a nationally representative survey of 72,250 adults aged 45 and above across all states and union territories of India. LASI is envisioned to be conducted every two years for the next 25 years^35^.

The LASI survey is conceptually comparable to the United States Health and Retirement Study (HRS) and other HRS-type surveys in various countries, including China (China Health and Retirement Longitudinal Survey) and England (English Longitudinal Study of Ageing)^35^. Along with its uniqueness of comparability with studies in other countries, LASI also considered features unique to India, including its institutional and cultural characteristics. LASI is conducted through a partnership of the International Institute of Population Sciences (IIPS), Harvard University, and the RAND Corporation^35^. The Indian Council of Medical Research (ICMR) extended the necessary guidance and ethical approval for conducting the LASI. All the participants were provided with information brochures explaining the purpose of the survey, ways of protecting their privacy, and the safety of the health assessments. As per the ethics protocol, the consent forms were administered to each participant.

The LASI survey protocol utilizes state-of-the-art technology in sample management interviewing, Computer-Assisted Personal Interviews (CAPI), and data processing. This protocol helps in releasing data faster and ensures data quality through built-in checks in CAPI and real-time data monitoring with an automated data quality control protocol. During the pilot survey conducted in 2010, LASI developed an automated quality control protocol, building on the Sample Management Software (SMS) and CAPI technologies associated with RAND’s information system, MMIC™ (Multimode Interviewing Capability). CAPI directly records the responses of survey participants. This method requires field teams to be outfitted with laptop computers pre-loaded with survey questions asked of respondents in a face-to-face interview. The field teams directly store the data into a laptop computer, which minimizes the data recording and entry errors. The full-scale use of this protocol allows the monitoring of fieldwork progress and checks the quality of collected information by examining data (e.g., for internal inconsistencies and missingness) at the level of interviewers and state fieldwork agencies. Since we are interested in exploring the physical activity-cognitive health association among older individuals, we restrict our attention to the subsample respondents aged 60 years or above. The present study dataset contains 31,464 respondents, 16,366 women, and 15,098 men.

### Variable description

#### Outcome variable

Cognitive impairment was measured through five broad domains (memory, orientation, arithmetic function, executive function, and object naming)^35^. Memory was measured using immediate word recall, delayed word recall; orientation was measured using time and place measure, arithmetic function was measured through backward counting, serial seven, and computation method; executive function was measured through paper folding and pentagon drawing method, and object naming was lastly done to measure the cognitive impairment among older adults. A composite score of 0–43 was computed using the domain-wise measure^35^.

### Treatment variable

Frequent vigorous physical activity status was coded as yes (every day) and no (more than once a week, once a week, one to three times in a month and never). The question through which physical activity was assessed was “How often do you take part in sports or vigorous activities, such as running or jogging, swimming, going to a health centre or gym, cycling, or digging with a spade or shovel, heavy lifting, chopping, farm work, fast bicycling, cycling with loads”?^35^. The vigorous physical activity questions were used among older adults in previous studies in India and other countries^36–38^.

### Explanatory variables

Aged was coded as young old (60–69 years), old-old (70–79 years) and oldest old (80 + years). Educational status was coded as no education/primary not completed, primary, secondary and higher. Marital status was coded as currently married, widowed and others. Others included divorced/separated/never married. Living arrangement was coded as living alone, living with spouse, living with children and others. Working status was coded as never worked, currently working, currently not working and retired. Social participation was coded as no and yes. Respondents were said to be socially engaged if they participate in the following activities. Eat out of house (Restaurant/Hotel); Go to park/beach for relaxing/entertainment; Play cards or indoor games; Play out door games/sports/exercise/jog/yoga; Visit relatives/friends; Attend cultural performances/shows/Cinema; Attend religious functions /events such as bhajan/satsang/prayer; Attend political/community/organization group meetings; Read books/newspapers/magazines; Watch television/listen radio and use a computer for e-mail/net surfing etc. If the respondent was involved in any of the above activity, then the respondent was defined to be socially engaged. Obesity/overweight were coded as no and yes. The respondents with body mass index (BMI) more than 25 was coded as obese/overweight. Alcohol and tobacco consumption was coded as no and yes.

The probable major depression among the older adults with symptoms of dysphoria, calculated using the CIDI-SF (Short Form Composite International Diagnostic Interview) score of 3 or more^39^. This scale estimates a probable psychiatric diagnosis of major depression and has been validated in field settings and widely used in population-based health surveys. The lowest 10th percentile is used as a proxy measure of severe depression among older adults^35,40,41^. Self-rated health was coded as good which includes excellent, very good, and good whereas poor includes fair and poor. Difficulty in ADL (Activities of Daily Living) was coded as no and yes. Activities of Daily Living (ADL) is a term used to refer to normal daily self-care activities (such as movement in bed, changing position from sitting to standing, feeding, bathing, dressing, grooming, personal hygiene, etc.). The ability or inability to perform ADLs is used to assess a person's functional state, particularly for persons with impairments and those who are older^42^. If the respondent reported any difficulty in above ADLs, then difficulty in ADL was coded as yes otherwise no. Difficulty in IADL (Instrumental Activities of Daily Living) was coded as no and yes. Instrumental activities of daily living that are not necessarily related to the fundamental functioning of a person, but they let an individual live independently in a community. These tasks are necessary for independent functioning in the community. Respondents were asked if they were experiencing any difficulties that were expected to last longer than three months, such as preparing a hot meal, shopping for groceries, making a phone call, taking medications, doing housework or gardening, managing money (such as paying bills and keeping track of expenses), and trying to navigate or finding an address in unfamiliar places^43^. If the respondent reported any difficulty in above IADL’s then difficulty in IADL was coded as yes otherwise no. Morbidity status was categorized as 0 “no morbidity”, 1 “any one morbid condition” and 2+ “co-morbidity”^44^.

Using household consumption data, the monthly per-capita consumption expenditure (MPCE) quintile was determined. The sample households were canvassed using sets of 11 and 29 questions on food and non-food expenses, respectively. Food spending was gathered over a seven-day reference period, whereas non-food expenditure was collected over 30-day and 365-day reference periods. The 30-day reference period has been used to standardise food and non-food expenses. The monthly per capita consumption expenditure (MPCE) is calculated and used to summarise consumption^35^. The variable was then divided into five quintiles i.e., from poorest to richest. Religion was coded as Hindu, Muslim, Christian, and Others. Caste was recoded as Scheduled Tribe, Scheduled Caste, Other Backward Class, and others. The Scheduled Caste includes a group of the population that is socially segregated and financially/economically by their low status as per Hindu caste hierarchy. The Scheduled Tribes (STs) and Scheduled Castes (SCs) are among the most disadvantaged and discriminated socio-economic groups in India. The OBC refers to persons who have been classified as "educationally, economically, and socially backward." The OBCs are considered lower castes in the old caste system, although they are not untouchables. The “other” caste category is identified as having higher social status. Place of residence was coded as rural and urban. The regions of India were coded as North, Central, East, Northeast, West, and South.

### Statistical analysis

In the present study, we used propensity score matching (PSM) to assess the treatment effects of physical activity on cognitive functioning. PSM is a statistical technique that minimizes selection bias, mimics the experimental design with balanced baseline characteristics, and helps evaluate the treatment effects for observation/cross-sectional data. It creates a comparison group that can address the counterfactual (defined as what would be the outcome if the treatment did not occur). In PSM, various observed predictors are used to create a propensity score that indicates each person's probability to be included in the treatment group. This score is then used to create a matched sample of treatment and control participants^45^. The propensity score is a balancing score of the observed predictors, indicating that the covariates' distribution is similar for the treatment and comparison groups^46^. The individuals who were frequently physically active were assigned to the treatment group and matched with the control group using a one-to-one matching method. The calculated propensity scores were based on various individual and household-level characteristics, including age, educational status, working status, marital status, living arrangement, social participation, overweight/obese, alcohol and tobacco consumption, self-rated health, depression, difficulties in ADL and IADL, multi-morbidity, MPCE quintile, religion, caste, residence, and region.

We further calculated Average Treatment Effect on the Treated (ATT) that is defined as the average increase in the cognitive functioning of those who were frequently physically active, as compared to those who were not frequently physically active and had similar background characteristics (by matching)^47^. The Average Treatment Effect on the Untreated (ATU) was calculated as the average increase in the cognitive functioning of those who were not frequently physically active compared to those who were frequently physically active and had similar background characteristics (by matching). For propensity score matching (PSM) analysis, we used the "*psmatch2*" command in Stata (version 14.1)^48^. In addition, the model adequacy of PSM was assessed by the Mantel–Haenszel sensitivity analysis. We used "*mhbounds*" command, a Stata program to check if the ATT estimate of a given binary outcome was susceptible to a possible hidden bias caused by unobserved confounders. A Gamma value of 1 means the ATT estimate was free of hidden bias^49^.

We used multiple linear regression analysis to assess the associations between physical activeness and cognitive functioning after adjusting for various individual and household factors. This analysis consists of two parts. First, a linear regression model was built using the full sample (pre-matching). Second, after propensity score matching analysis, a linear regression analysis was performed using a reduced (post-matching) sample containing only those cases included in the matches.

### Ethics approval

The Central Ethics Committee on Human Research (CECHR) under the Indian Council of Medical Research (ICMR) extended the necessary guidance, guidelines and ethics approval for conducting the LASI survey. And all methods were carried out in accordance with those relevant guidelines and regulations.

### Consent to participate

The survey agencies that conducted the field survey for the data collection have collected prior informed consent (signed and oral) for both the interviews and biomarker tests from the eligible respondents in accordance with Human Subjects Protection.

## Results

Table 1 shows the socio-economic and demographic characteristics of the older adults (15,098 men and 16,366 women) included in the sample. The mean age of older males was 69.3 (CI 69.1–69.4) years whereas for older females it was 69.1 (69.0–69.2) years. Around 24.6 percent of older men and 12.0 percent of older women were frequently physically active. Women in the final sample were slightly younger than men. Compared to older men, a higher proportion of older women were uneducated (81.4% vs. 53.1%). Around four-fifths of older men (81%) and nearly two-fifth of older women (44.1%) were currently married. More than half of the older women (54.0%) were widowed. Relatively, older women were more likely to live alone (8.5% vs. 2.5%) than older men. Nearly 23.2 percent of older women and 15.5 percent of older men were overweight or obese. The alcohol (27.6% vs. 2.6%) and tobacco (59.0% vs. 22.4%) consumption were comparatively higher in older men than in older women. Regarding activities of daily living (ADL), women had greater difficulty with both basic ADL (26.5% vs. 21.9%) and instrumental ADL (56.9% vs. 39.7%) compared to men. Older women had a comparatively higher prevalence of depression (9.5% vs. 7.2%) and self-reported multi-morbidity (25.4% vs. 22.2%) than older men.

**Table 1:** Socio-economic profile of the study respondents, 2017–18.

Variables	Male		Female	
	Mean	CI	Mean	CI
Cognitive score	26.4	26.3–26.5	22.1	22.0–22.2
Age in years	69.3	69.1–69.4	69.1	69.0–69.2

	Sample	Percentage	Sample	Percentage
**Frequent physical activity**				
No	11,392	75.5	14,400	88.0
Yes	3706	24.6	1966	12.0
**Individual factors**				
Age				
Young-old	8730	57.8	9678	59.1
Old-old	4702	31.1	4803	29.4
Oldest-old	1666	11.0	1886	11.5
Education				
No education/primary not completed	8019	53.1	13,314	81.4
Primary	2235	14.8	1297	7.9
Secondary	3096	20.5	1297	7.9
Higher	1748	11.6	458	2.8
Marital status				
Currently married	12,242	81.1	7211	44.1
Widowed	2489	16.5	8837	54.0
Others	366	2.4	318	2.0
Living arrangement				
Living alone	380	2.5	1397	8.5
Living with spouse	3929	26.0	2485	15.2
Living with children and spouse	10,205	67.6	11,268	68.9
Living with others	583	3.9	1216	7.4
Working status				
Never worked	575	3.8	7662	46.8
Currently working	6348	42.1	3088	18.9
Currently not working	6172	40.9	5311	32.5
Retired	1999	13.3	302	1.8
Social participation				
No	1192	7.9	1722	10.5
Yes	13,906	92.1	14,644	89.5
Obesity/overweight				
No	12,755	84.5	12,568	76.8
Yes	2343	15.5	3798	23.2
Alcohol consumption				
No	10,939	72.5	15,943	97.4
Yes	4159	27.6	423	2.6
Tobacco consumption				
No	6197	41.1	12,706	77.6
Yes	8901	59.0	3660	22.4
**Health factors**				
Depression				
No	14,006	92.8	14,818	90.5
Yes	1092	7.2	1548	9.5
Self-rated health				
Good	8253	54.7	8335	50.9
Poor	6845	45.3	8031	49.1
Difficulty in ADL				
No	11,788	78.1	12,022	73.5
Yes	3310	21.9	4344	26.5
Difficulty in IADL				
No	9112	60.4	7047	43.1
Yes	5986	39.7	9319	56.9
Morbidity				
0	7507	49.7	7274	44.5
1	4240	28.1	4928	30.1
2 +	3351	22.2	4164	25.4
**Household factors**				
MPCE quintile				
Poorest	3145	20.8	3681	22.5
Poorer	3219	21.3	3611	22.1
Middle	3262	21.6	3331	20.4
Richer	2902	19.2	3136	19.2
Richest	2570	17.0	2607	15.9
Religion				
Hindu	12,386	82.0	13,484	82.4
Muslim	1769	11.7	1781	10.9
Christian	388	2.6	511	3.1
Others	555	3.7	590	3.6
Caste				
Scheduled caste	2836	18.8	3113	19.0
Scheduled tribe	1166	7.7	1389	8.5
Other backward class	6925	45.9	7308	44.7
Others	4172	27.6	4556	27.8
Place of residence				
Rural	10,879	72.1	11,322	69.2
Urban	4219	28.0	5044	30.8
Region				
North	1863	12.3	2096	12.8
Central	3395	22.5	3202	19.6
East	3713	24.6	3729	22.8
Northeast	437	2.9	497	3.0
West	2457	16.3	2941	18.0
South	3233	21.4	3900	23.8
**Total**	15,098	100	16,366	100

The percentage difference in the level of physical activity (frequent vs. not frequent) among older adults by gender is presented in Table 2. Men who were aged 70–79 years (old-old), widowed, living alone, not working, had poor self-rated health, difficulties in activities of daily living (ADL and IADL), and multi-morbidity were less frequently physically active than their respective counterparts. A similar pattern was found for older women, except for living arrangements where older women living with children and spouses were less frequently physically active than those living alone. According to household characteristics, the level of physical activity was lower among urban residents and poor older adults. Table 3 shows the matching analysis results for the effect of frequent physical activeness on cognitive functioning. The unmatched sample estimates for cognitive functioning among older men and women show that, on average, the cognitive function for those who were frequently physically active was increased by 0.73 and 1.07 points, respectively, compared to those who were not frequently physically active. The association between physical activeness and cognitive functioning was significant for older men and older women in the matched sample. Among older men, the cognitive functioning on average was increased by 0.75 points for those who were physically active than those who were not physically active but had similar background characteristics. On the other hand, the cognitive functioning among older women who were physically active was increased by 0.82 points than their counterparts with similar background characteristics.

**Table 2:** Percentage of older adults who were involved in frequent physical activity and not involved in frequent physical activity, LASI, 2017–18.

Variables	Male			Female		
	No frequent physical activity	Frequent physical activity	Difference	No frequent physical activity	Frequent physical activity	Difference
**Individual factors**						
Age						
Young-old	53.0	72.7	19.8	57.0	75.0	18.1
Old-old	33.8	22.8	− 11.0	30.6	20.3	− 10.3
Oldest-old	13.2	4.4	− 8.8	12.5	4.7	− 7.8
Education						
No education/primary not completed	52.5	54.9	2.4	81.4	81.0	− 0.4
Primary	14.8	14.9	0.1	7.8	8.9	1.1
Secondary	21.0	19.0	− 2.0	8.1	6.7	− 1.3
Higher	11.7	11.2	− 0.5	2.7	3.4	0.7
Marital status						
Currently married	79.1	87.1	7.9	42.2	57.8	15.6
Widowed	18.4	10.7	− 7.7	56.0	39.2	− 16.8
Others	2.5	2.2	− 0.3	1.8	3.1	1.3
Living arrangement						
Living alone	2.9	1.5	− 1.4	8.3	10.5	2.2
Living with spouse	26.3	25.2	− 1.1	14.5	20.2	5.7
Living with children and spouse	66.5	70.9	4.4	69.5	64.0	− 5.5
Living with others	4.3	2.4	− 1.9	7.7	5.3	− 2.4
Working status						
Never worked	4.4	2.1	− 2.2	49.9	24.0	− 25.9
Currently working	31.7	74.0	42.3	14.0	54.8	40.9
Currently not working	49.6	14.0	− 35.6	34.2	19.6	− 14.6
Retired	14.4	9.8	− 4.5	1.9	1.6	− 0.3
Obesity/overweight						
No	84.7	83.7	− 1.0	77.2	73.9	− 3.3
Yes	15.3	16.3	1.0	22.8	26.1	3.3
Alcohol consumption						
No	72.9	71.2	− 1.7	97.6	96.4	− 1.1
Yes	27.1	28.9	1.7	2.5	3.6	1.1
Tobacco consumption						
No	41.9	38.5	− 3.4	78.0	74.9	− 3.1
Yes	58.1	61.5	3.4	22.0	25.1	3.1
**Health factors**						
Self-rated health						
Good	52.1	62.5	10.4	48.9	65.8	16.9
Poor	47.9	37.5	− 10.4	51.1	34.2	− 16.9
Difficulty in ADL						
No	75.6	85.8	10.2	72.5	80.8	8.4
Yes	24.4	14.2	− 10.2	27.6	19.2	− 8.4
Difficulty in IADL						
No	57.4	69.4	12.0	42.2	49.0	6.8
Yes	42.6	30.6	− 12.0	57.8	51.0	-6.8
Morbidity						
0	47.0	58.1	11.1	44.0	47.7	3.6
1	28.9	25.5	− 3.4	29.5	34.3	4.8
2 +	24.1	16.4	− 7.7	26.5	18.0	− 8.5
**Household factors**						
MPCE quintile						
Poorest	21.0	20.2	− 0.8	23.0	19.1	− 3.9
Poorer	21.6	20.6	− 1.0	22.1	21.8	− 0.3
Middle	21.0	23.5	2.5	20.1	22.3	2.2
Richer	18.9	20.1	1.2	18.9	21.3	2.4
Richest	17.5	15.6	− 1.9	16.0	15.5	− 0.4
Religion						0.0
Hindu	81.3	84.3	3.0	81.9	86.3	4.4
Muslim	12.2	10.2	− 2.1	11.4	7.0	− 4.5
Christian	2.7	2.2	− 0.5	3.2	2.4	− 0.8
Others	3.8	3.4	− 0.4	3.5	4.4	0.9
Caste						
Scheduled caste	18.6	19.3	0.7	19.2	17.8	− 1.4
Scheduled tribe	7.4	8.7	1.3	7.9	12.5	4.6
Other backward class	45.1	48.3	3.2	44.3	47.5	3.2
Others	28.9	23.8	− 5.1	28.6	22.2	− 6.4
Place of residence						
Rural	70.4	77.2	6.8	68.8	72.2	3.4
Urban	29.6	22.8	− 6.8	31.2	27.8	− 3.4
Region						
North	13.4	8.9	− 4.5	13.2	10.0	− 3.2
Central	23.1	20.7	− 2.3	20.4	13.8	− 6.5
East	25.3	22.3	− 3.1	24.0	13.8	− 10.2
Northeast	2.7	3.4	0.6	3.1	2.9	− 0.2
West	14.4	22.0	7.6	16.3	30.3	14.0
South	21.0	22.7	1.7	23.1	29.2	6.1
**Total**	100	100	0.0	100	100	0.0

Differences: Frequent physical activity − No frequent physical activity.

**Table 3:** Results of matching estimates showing the effect of frequent physical activity on cognitive scores among older adults, LASI, 2017–2018.

	Treated	Control	Differences	SE	p-value
**Male**					
**Frequent physical activity**					
Cognitive score (0–43)					
Unmatched	27.346	26.617	0.728	0.126	
ATT	27.346	26.592	0.754	0.180	0.001
ATU	26.617	27.680	1.063		
ATE			0.986		
**Female**					
**Frequent physical activity**					
Cognitive score (0–43)					
Unmatched	23.594	22.522	1.072	0.179	
ATT	23.594	22.774	0.821	0.251	0.001
ATU	22.538	23.935	1.397		
ATE			1.323		

The model was controlled for individual, health and household factors.

The average treatment effect (ATE) estimates the average increase in cognitive functioning if the whole population were physically active. The cognitive function was increased by 0.98 and 1.32 points for the frequently physically active older men and older women population, respectively. Although physical activeness positively affects cognitive functioning, the effect was slightly higher in older women than in older men. Average Treatment Effect on the Untreated (ATU) results indicated that among those who were not physically active if they were physically active, the average cognitive function was likely to increase by 1.06 and 1.39 points for older men and older women respectively.

Table 4 showed the adjusted regression coefficients for cognitive functioning by gender for unmatched and matched (PSM) samples. In the unmatched sample, after adjusting for various individual, health, lifestyle, and household factors, results suggest that physical activeness was significantly associated with greater cognitive functioning for both older men (β = 0.73; 95% CI 0.51, 0.96) and older women (β = 1.09; 95% CI 0.77, 1.40). Similar results were observed in the matched sample.

**Table 4:** Adjusted regression coefficients (for cognitive scores)—unmatched and PSM models.

Outcome variable (cognitive score)	Unmatched	PSM	
		ATE	ATT
**Male**			
Frequent physical activity (Yes vs no)	0.73* (0.51–0.96)	1.22* (0.92–1.52)	0.71* (0.39–1.18)
**Female**			
Frequent physical activity (Yes vs no)	1.09* (0.77–1.40)	1.29* (0.83–1.76)	0.74* (0.31–1.18)

The model was control for individual, health and household factors.

**Figure 2:**
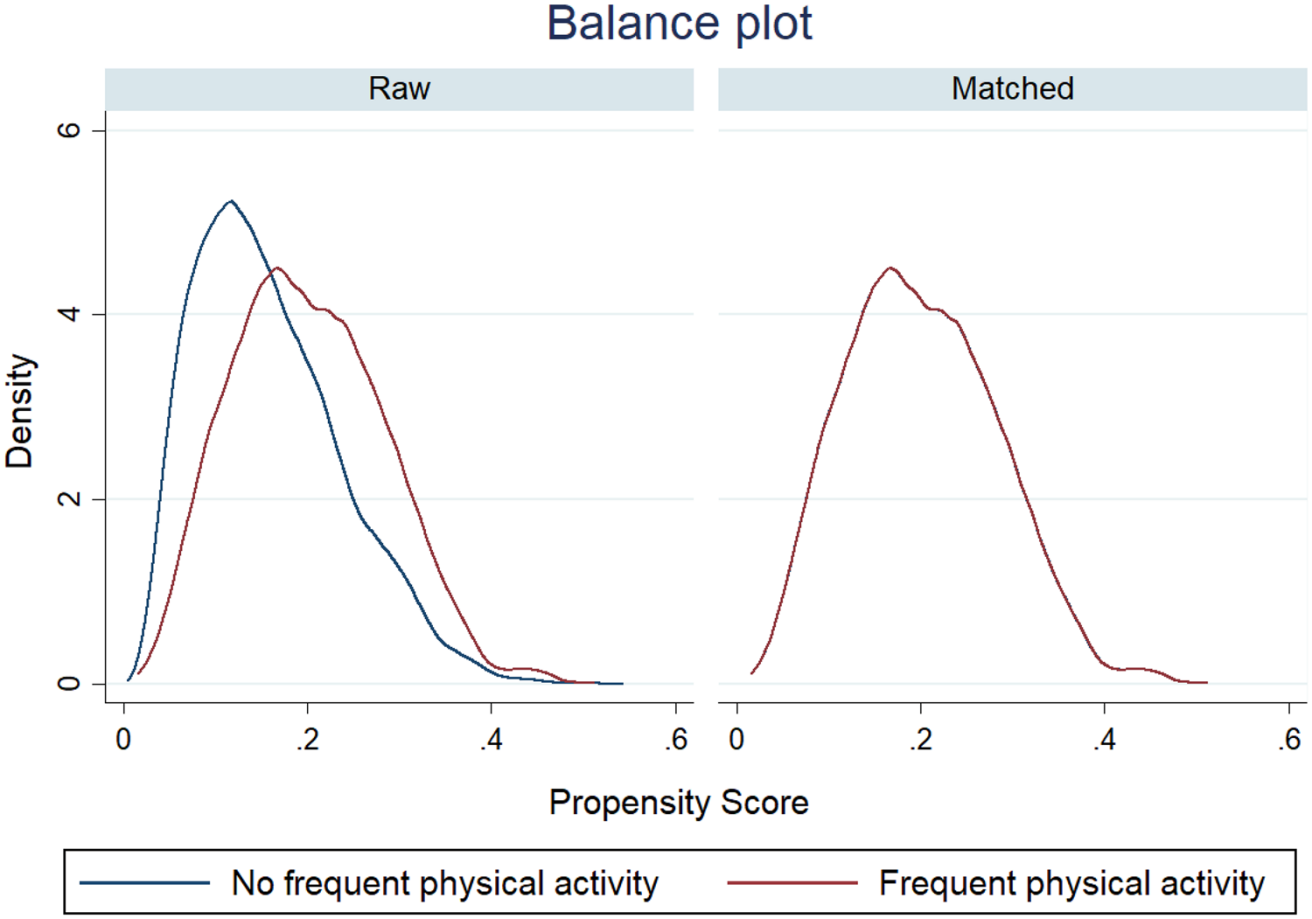
Balance plot before and after propensity matching.

**Figure 3:**
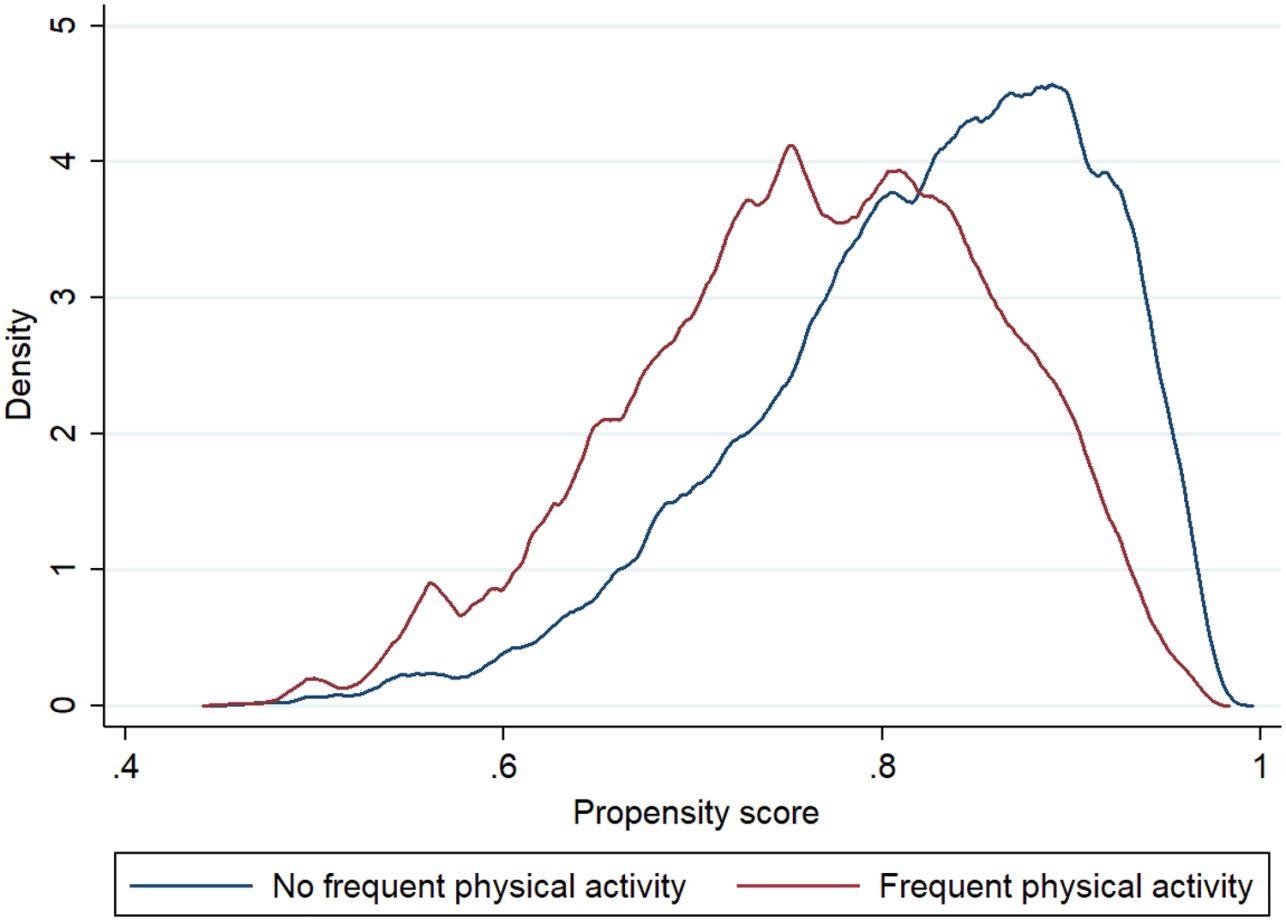
Overlap plot for the propensity score of each treatment.

The balance plot is the kernel density plots of the covariates over treatment levels for raw and matched samples. The balance plot in Fig. 2 shows that both control and treatment groups were balanced. According to the overlap assumption, each individual has a positive probability of receiving each treatment level. The overlap plot in Fig. 3 depicts that neither too much probability mass near zero nor one and most of the masses for these two estimated densities lie in regions where they overlap each other. Thus, we can conclude that there is no evidence of a violation of the overlap assumption. In our analysis, satisfying covariate balance and overlap assumptions indicate the unbiasedness in the estimated treatment effects.

## Discussion

This study aimed to investigate the association of physical activity with cognitive functioning in older adults with a gender lens by utilising the PSM method of estimating the impact of a specific intervention. To our knowledge, this study was the first to have added empirical evidence regarding gender differences in cognitive functioning among older Indian adults considering their physical activity status by constructing an artificial control group.

A proportion of 24.6% of older men and 12.0% of older women in the current study were found to be frequently physically active. The higher prevalence of vigorous physical activities in older adults may be explained by the definition of physical activity (vigorous) in this paper which includes the farm-related activities. A large proportion of older population in rural areas of the country are engaged in agricultural labor^50^. Another study based on the data from the LASI pilot survey, using the same definition of vigorous activity, has estimated that 27% of the participants were engaged in vigorous activities^37^. On the other hand, the significant gender difference in the prevalence of physical activity may be attributed to the fact that traditionally, in India, men have been considered as income earners while women are confined to the household. Similarly, studies based in other developing countries reported gender differences in the prevalence of vigorous physical activities^51,52^. The previous study reported that the prevalence of vigorous activities during the farming season was nearly 33% and highlighted the existing gender differences by reporting that vigorous activities among the male farmers were greater than female farmers irrespective of the season of farming^52^.

Our results suggest that older adults who engaged in physical activity have higher cognitive functioning score compared with older adults without physical activity. The finding is consistent with previous reviews of observational studies that have concluded that interventions that promote higher levels of physical activity in old age are associated with a slower rate of cognitive decline^14,53,54^. Evidence also suggest that regular physical activity through management of cardiovascular risk factors such as hypertension, diabetes and obesity may protect the cognitive abilities and reduce the risk of late-life dementia^55^. Several studies based on cross-sectional/ prospective cohort design as well as randomized controlled trials (RCTs) have shown similar findings that suggest a significantly larger hippocampal or grey matter volumes and associated better spatial memory and improved cognitive functioning among physically fit older individuals compared with their unfit counterparts^56,57^. Hence, exercise and physical activity interventions for older adults may help ameliorate age-related deficits in cognitive functioning by providing a better executive functioning and appropriate decision making, that in turn result in avoiding adverse life habits in later years of life.

However, it is also plausible that a reciprocal association is present, such that remaining cognitively active may positively influence being physically active. In particular, this effect might occur through higher performance in executive functioning that have a positive impact on independence and functionality among older adults as shown in the previous studies^58–60^. Again, as documented, further cognitive deficits could possibly be explained by the decline in physical activity in people in later years of life with cognitive impairment or with lower score in cognitive functioning. On the other hand, multiple studies have found no evidence of slowing down the cognitive decline in people doing more physical activity^61–65^. Another study suggests that although physical activity levels were found lower in the years leading up to diagnosis of dementia, such a reduction in physical activity might simply be a part of preclinical symptoms of dementia^66^. Nevertheless, by using the PSM method in the current study which mimics the experimental design with balanced baseline characteristics, a potential positive causal association between physical activities and cognitive functioning among Indian older adults is suggested.

Another important finding of our study is that after including for critical confounding factors, such as socio-demographics and health behaviours, women had a stronger cognitive score compared to men while they were assumedly treated with physical activity. Recent studies showed that women’s lower cognitive functioning is importantly explained by the individual attributes such as differences in educational and occupational levels and poor health status^67–69^. Moreover, in comparison to men, a shorter duration of formal education, longest occupation being domestic worker and the psychiatric disorders associated with widowhood and lack of social resource accounted for steeper cognitive decline in women than in men^70–73^. Hence, the increased physical activity in women might have a stronger influence on their cognitive abilities. Similarly, the longer life expectancy of women could also explain observed gender differences in the association between physical activity and cognitive functioning Women have a larger burden of impairment than men^74,75^. Thus, as the current results suggest the interventional programs that focus on promoting physical activity in old age may have greater impact on cognitive health especially among women. Furthermore, women’s increased engagement in group activities and social interactions that have beneficial effects on their brain volume and executive functioning may reduce the gender gap observed in cognitive functioning^15^.

There are several limitations to this study. Importantly, the study is conducted with a cross-sectional design and relies upon self-report for physical activity. Also, the possibility of unobserved confounding factors may exist since the method of PSM is limited by the fact that it can only control for observed confounders. This again suggests the need for future longitudinal studies to test the current conclusions. Although the cognitive functioning module was based on multiple domains and was multi-faceted, mechanisms underlying the physical and executive functioning that directly affect cognitive domains are not taken into account in the study. Further, finding of the relationship of physical activity and cognitive functioning is often attributed to the underlying neurobiological differences between older adults with cognitive deficits. For example, compared to cognitively healthy older adults, those with mild cognitive impairment have greater amounts of beta-amyloid accumulation; accelerated atrophy in the medial temporal lobe; and decreased connectivity of the hippocampus with the brain^76–79^. These underlying changes in the brain may potentially alter the relationships of behavioural factors with cognitive functioning, leading to an attenuation of the relationship between physical activity and cognitive functioning observed in our study. This reveals the need for further investigation with more clinical information as well as longitudinal design.

Nevertheless, the findings of our study have important public health implications because the analyses are conducted in a large sample of older adults derived from a nationally-representative survey, and explored the treatment effects of physical activity on cognitive functioning with comparing to an artificial control group that is untreated with such intervention, which in turn provides a potential causal effect of such treatment. The data also provided exhaustive and comprehensive information on aging population by which the major confounding factors could be controlled throughout the analyses.

## Conclusion

Given rapid increase in life expectancy, cognitive disability is increasingly a public health challenge. The findings of the present study suggest physical activity as one of the modifiable risk factors that prevent or delay the onset of cognitive impairment. Owing to its cardio-protective effect, physical activity can be considered as a potential factor that stimulates the brain activities and cognitive functioning in old age and can be accommodated in interventions related to active aging. Therefore, health practitioners aiming to improve the cognitive functioning of older patients or clients might benefit from the development of the interventions related to physical activities designed to reduce the decline of cognitive resources. Moreover, in terms of cognitive enrichment in older population and in women in particular by initiating behavioural interventions that can also contribute to optimizing a successful aging, further longitudinal studies are warranted.

## Data Availability

The study uses secondary data which is available on reasonable request through https://www.iipsindia.ac.in/content/lasi-wave-i.
